# Effect of Two Feed Additives—One Multicomponent Based on Nanosilica and the Second Containing Mycelium of *Lentinula edodes* Fortified with Selenium—On Production Parameters and Histological Analysis of Calves’ Duodenum and Abdominal Rumen

**DOI:** 10.3390/ani12101246

**Published:** 2022-05-12

**Authors:** Dorota Bederska-Łojewska, Bożena Muszyńska, Sylwia Orczewska-Dudek, Marian Kamyczek, Ewelina Kmiecik, Jan Lazur, Marek Pieszka

**Affiliations:** 1 Department of Animal Nutrition and Feed Science, National Research Institute of Animal Production, ul. Krakowska 1, 32-083 Balice, Poland; sylwia.orczewska@iz.edu.pl (S.O.-D.); marek.pieszka@iz.edu.pl (M.P.); 2 Department of Neurobiology, Institute of Pharmacology, Polish Academy of Sciences, Smętna 12, 31-343 Kraków, Poland; 3 Department of Pharmaceutical Botany, Jagiellonian University Collegium Medicum, Medyczna Street 9, 30-688 Cracow, Poland; muchon@poczta.fm (B.M.); janlazur@gmail.com (J.L.); 4 The Institute’s Experimental Station, National Research Institute of Animal Production, Mielzynskich Street 14, 64-122 Pawlowice, Poland; marian.kamyczek@zdpawlowice.pl (M.K.); ferma.bydla@zdpawlowice.pl (E.K.)

**Keywords:** mushroom, average daily feed intake, average daily gain, feed conversion ratio, hematological and biochemical parameters

## Abstract

**Simple Summary:**

In the current study, we investigated the effects of two different additives on calves’ production parameters (body weight, average daily gain, average daily feed intake, and feed conversion ratio), hematological and biochemical results; serum selenium level, and histology of duodenum and rumen. A total of 18 1-day-old male calves were randomly assigned to control and two experimental treatments for 70 days. The first additive contained nanosilica with pancreatic enzymes, a mixture of fat-coated organic acids (fumaric, malic, citric, and sorbic acids), and sodium butyrate, whereas the second was based on the mycelium of *L. edodes* enriched in selenium. At the end of the experiment, three animals from each group were sacrificed for histological analysis of the digestive tract (abdominal rumen and small intestine). The findings of this study indicate that supplementation had no influence on growth performance, nor hematological or biochemical parameters. The additives did not increase duodenal crypt depth, villi height or width, or rumen papillae height or width. In the study, we observed higher levels of serum selenium and lower average daily milk replacer intake at 35–42 d but higher levels from 42 to 70 d in the animals receiving the inclusion of modified *L. edodes* mycelium.

**Abstract:**

With this study, we aimed to evaluate the effect of two multicomponent feed additives given to animals from 10 to 70 d with milk replacer on the production parameters of reared calves: serum selenium level and histology of duodenum and rumen. The first additive was based on nanosilica (3000 mg) containing pancreatic enzymes (protease (18 mg), lipase (45 mg)), a mixture of fat-coated organic acids (2000 mg) (fumaric, malic, citric, and sorbic acids), and sodium butyrate (10,000 mg) (nanosilica/E/OA/SB), whereas the second was based on the mycelium of *L. edodes* (7 g) enriched in selenium (0.058 mg/g d.w.) (*L. edodes*/Se). The study was conducted on 18 male crossbred Holstein dairy calves from birth to 70 days of age, which were randomly assigned to control and experimental groups (six animals in each). From each group, three animals were sacrificed at 70 days of age, and histological analysis of the digestive tract (abdominal rumen and small intestine) was performed. It was observed that the additives used did not have any effect on growth performance and hematological or biochemical parameters. However, higher levels of selenium were found in serum in the calves fed with modified *L. edodes* mycelium on days 35 and 70 (44.33 and 51.33 µg/L in the control group and 132 and 93 µg/L in the *L. edodes*/Se group, respectively; *p* < 0.001). Moreover, we noticed lower average daily milk replacer intake at 35–42 d, which increased significantly from 42 to 70 d in the animals receiving *L. edodes/Se*.

## 1. Introduction

Diarrhea and pneumonia are the main reasons for calf falls in the early weeks of life. Scientific data indicate that the rate of animal mortality due to gastrointestinal (GI) tract disorders is 1.1%. In light of the ban on antibiotic growth promoters, it is necessary to look for natural feed additives that can improve the growth performance of animals and help to maintain the homeostasis of intestines [1].

Both organic and inorganic acids, as well as their salts, play an important role in animal nutrition and may be used as potential alternatives to antibiotics in animal diets [2,3]. Sufficiently low acidity of the GI tract stabilizes the bacterial flora and prevents the overgrowth of unwanted bacteria (e.g., enteropathogenic *E. coli*). Lower pH of the digestive tract, especially in young animals, activates pepsinogen and stimulates the production of bicarbonates [3,4]. The encapsulation of organic acids in the lipid matrix eliminates some unfavorable properties of nonencapsulated acidifiers. They have no effect on the pH of forage and do not inhibit the secretion of gastric juice like classic acidifiers. A great advantage of encapsulated acidifiers is that they are released along the entire length of the small intestine [5].

Research shows that calves are characterized by an innate weaning rhythm, the related process of development of the rumen and forestomachs, and the decrease in exocrine pancreatic function [6]. For this reason, one hypothesis assumes that supplementing the diet of a young calf with pancreatic enzymes could improve the digestion of the ration components. Microbial enzymes have been used in experiments to support the secretory function of the pancreas and improve digestion.

Studies have shown that butyric acid is essential for the development of enterocytes. A study by Niwińska [7] indicated that the addition of butyrate may speed up development of the GI tract, allowing for better digestion of nutrients. Sodium butyrate belongs to a group of compounds called volatile fatty acids (VFA), which accelerate the development of rumen by promoting papillae growth and improving calf metabolism [8].

Multicomponent feed additives that can offer a broader spectrum of activity are gaining attention in animal nutrition. Currently, there are no literature reports describing the effect of silicon nanoparticles on the development of young calves. Existing scientific reports confirm the positive effects of silicon or silver nanostructures on animal development. Their beneficial action on the weight gain of animals is mainly attributed to the improvement of the microbiological status of the intestines and better feed use [9,10]. An experiment carried out by Szczurek [11] using silica nanostructures in young piglets showed that the additive improved the production parameters, including body weight gain (BWG) and feed intake (FI).

Because many areas of the earth are deficient in selenium, its concentration and biological availability in the soil affect its content in plants and animal products. In the case of feeding calves, attention should be paid to the forage supplied, as the animals are born with low levels of this essential micronutrient. The most popular method of preventing selenium deficiency is administering a selenium injection in the initial days of life [12,13]. This essential trace mineral plays a key role in immune functions and animal health. Insufficient Se intake may result in decreased activity of antioxidant enzymes. T lymphocytes, the cell membranes of which contain lipids that are more prone to oxidation than the lipid membranes of B lymphocytes, are particularly more sensitive to Se deficiency [14]. Selenium deficiency can cause many adverse changes in the immune system and lead to myodegeneration, a condition called “white muscle disease” [15].

*Lentinula edodes* (shiitake) is one of the most popular edible/medicinal mushrooms. The polysaccharides found in the fruiting bodies of *L. edodes* can enhance the immune system and possess strong anticancer, antiviral, and antibacterial properties [16,17,18,19]. Moreover, *L. edodes* is a good source of vitamins and can accumulate bioelements [20,21,22].

The research hypothesis based on the literature mentioned above assumes that the administration of additives containing silicon nanostructures, enzymes, organic acids, sodium butyrate, or *L. edodes* cultures enriched with selenium to calves in the early rearing period will influence the development of the gastrointestinal tract and production indicators (body weight, body weight gain, and feed conversion ratio) and will prevent serum selenium deficiency.

In the present study, we analyzed the effects of two feed additives, the composition of which was based on substances that have a positive effect on the digestive tract. The first additive (nanosilica/E/OA/SB) was based on fat-coated organic acids, pancreatic enzymes, sodium butyrate, and nanosilica particles, whereas the second consisted of *L. edodes* mycelium enriched in selenium (*L. edodes*/Se). The aim of this study was to evaluate the impact of these two multicomponent preparations on the production parameters of reared calves.

## 2. Materials and Methods

### 2.1. Animals and Treatments

The trial was conducted on 18 crossbred (Polish Black and White (BW) × Holstein Friesian) male buffalo calves aged 0–70 days (six calves were sacrificed on day 70). The animals were kept at the Pawlowice Experimental Station in Poland.

All calvings took place over a period of 20 days to minimize the influence of seasons, temperature, and the resulting differences in metabolic processes, including the differences in thyroid gland activity in calves born in different months. The animals were randomly assigned to one of the groups after birth: control group, group receiving nanosilica/E/OA/SB (15,063 mg) (Table 1), and group receiving 7 g of *L. edodes* enriched in selenium (0.058 mg/g d.w.). The culture of mushroom–selenium additive was described previously by Muszyńska et al. (2020). The additives were given to the animals from 10 to 70 days with milk replacer (Sprayfo Violet, TROUW NUTRITION, Poland) prepared by mixing with water in a ratio of 1:7 (Table 2). For the first 7 days after delivery, the calves were kept in the delivery room in an individual cage lined with straw at a temperature of 17–25 °C. In the initial 4 days, the animals were fed with colostrum. The first feeding was up to 2 h after birth, with a minimum volume of 3 L. The calves were then fed twice a day with 2 L of colostrum per feeding. Later, the calves were given milk replacer in buckets twice a day. Starter (Fodder Industry in Pawlowice) ration was given starting on day 14 in the morning, and the leftover was weighed the next day to determine the feed intake of animals and feed conversion ratio (FCR). Natural (daytime) lighting in the livestock room was provided by windows with a glass-to-floor ratio of 1:20 and 1:18. The calves had free access to water and were vaccinated against bovine herpes virus type 1 (MSD Animal Health). The calves were housed, fed, and managed according to accepted standards of husbandry, hygiene, nutrition, and welfare. Table 3 shows the nutrient composition of the starter diet.

### 2.2. Measurements

The BW of calves was recorded weekly since birth before feeding, and feed intake was recorded daily. All calves were under clinical observation throughout the study.

### 2.3. Biochemical and Hematological Analyses and Serum Selenium Concentration Determination

Blood samples were collected from the external jugular vein of all calves at 10, 35, and 70 days of age for hematological (mean corpuscular volume (MCV), mean corpuscular hemoglobin (MCH), mean corpuscular hemoglobin concentration (MCHC), erythrocytes, hematocrit, hemoglobin, leukocytes, thrombocytes, cell distribution width (RDW), and mean corpuscular volume (MPV)) and biochemical analyses (alanine transaminase (ALT) and aspartate transaminase (AST)) and for determination of selenium concentration. Hematological analyses were carried out using a HORIBA SCIL VET ABC instrument (Horiba Medical, USA) immediately after the blood samples were collected. For the assessment of biochemical parameters (ALT, AST, and selenium level), blood was collected into heparinized tubes and later centrifuged at 5000× *g* rpm for 10 min at room temperature. The obtained plasma was divided into Eppendorf tubes for individual analyses and frozen at −20 °C. Biochemical analyses of plasma parameters were performed on a Mindray BS-180 (China) apparatus using commercial CORMAY kits (Lublin, Poland). The selenium level was tested by IDEXX laboratory (Kornwesthei, Germany) using an ICP-MS analyzer with an Se-78 isotope.

### 2.4. Histological Analysis

On the 70th day of life, three individuals from each group were euthanized, a fragment of the duodenum and the abdominal rumen (2 × 2 cm) was collected, and the length of the intestines was measured. The collected tissue fragments were rinsed with a physiological saline solution and fixed in 10% buffered formalin. Subsequently, the tissues were dehydrated in a Thermo Scientific Cytadel 2000 (USA) tissue processor by immersing in ethanol and xylene solutions of increasing concentrations. Then, the tissue fragments were infused with paraffin at 56 °C to sequentially fix them in paraffin blocks using a Myr EC 350-1 paraffin-embedding center with a Myr EC 350-2 cryo console. The paraffin blocks were cut into 5-µm-thick sections using a Thermo Scientific Microm HM 340E rotary microtome (Germany). The prepared material was transferred to a DiaPath (Italy) water bath at a temperature of 46 °C. After straightening, the material was transferred to a glass slide, which was then placed on a heating plate to allow the tissues to stick to the slide surface. The slides were immersed in xylene and placed in an alcohol series comprising 100%, 96%, and 75% ethanol. The prepared slides were then placed in distilled water and stained with hematoxylin and eosin. Later, they were rinsed in distilled water again and dehydrated in 75%, 96%, and 100% alcohol. Finally, the slides were dipped in xylene and covered with a cover slip using Canadian balm. All the slides were assessed under a Zeiss Axio microscope (Germany). The morphometric analysis included measurements of the height and width of the villi and the depth of the duodenal crypts (performed in 20 replications), as well as the length and width of the abdominal rumen papillae (eight measurements per individual).

### 2.5. Encapsulation of Organic Acids in a Triglyceride Matrix

The weighed amount of acids (fumaric, malic, citric, and sorbic acids) was encapsulated in a triglyceride matrix using a mixture of C12–C22 fatty acids in the form of triglycerides (Berg+Schmidt, Germany) and calcium sulfate (Chempur, Poland). The triglycerides were dissolved in a ceramic pot in a water bath at 80 °C; then, a mixture of calcium sulfate and fumaric, malic, citric, and sorbic acids was added to a final volume of 4% and mixed thoroughly. Fumaric, malic, citric, and sorbic acids accounted for 76% of the mixture volume. After mixing, the pot with the mixture was placed in a refrigerator at −20 °C for 60 min. Next, the mixture was ground in a laboratory mill with a 1.5 × 1.5 mm mesh diameter.

### 2.6. Nanosilica

Nanosilica was obtained by the pyrogenic method, which is used in the pharmaceutical industry. Silicon nanoparticles (Aerosil 200) used in the experiment were bought from Evonik (Germany). The nanosilica constituted hydrophilic fumed silica with a surface area of 200 m^2^/g and pH value between 3.7 and 4.5.

### 2.7. Enzymes and Sodium butyrate

Enzymes (Amano Enzyme Inc., Nagoya, Japan) were produced by *Aspergillus melleus* fermentation with an activity not less than 22.000 Ph.Eur. U/g for lipase AS and 24.000 Ph.Eur. U/g for protease N. Sodium butyrate was purchased from Nutri-Ad International NV (Turnhout, Belgium).

### 2.8. In Vitro Culture of Selenium-Enriched Mushroom Material

Fresh fruiting bodies of *L. edodes* (Berk.) Pegler from the *Omphalotaceae* family were used as the starting material for in vitro culture. Taxonomic identification was performed by Professor Bożena Muszyńska using the MycoKey 4.1 key (http://www.mycokey.com; accessed on 2 March 2018). Explants (fragments of mycelium) from the hymenial part of young fruiting bodies were degreased with 70% ethyl alcohol within 5 s and sterilized for 30 s in 15% HClO. After rinsing three times with redistilled water, sterile fruiting fragments were transferred under laminar airflow to Oddoux solid medium (agar-solidified) [22,24]. To obtain biomass for experiments, mycelia from solid in vitro cultures were inoculated on a modified liquid medium according to Oddoux. For this purpose, the biomass obtained from in vitro cultures on the solid medium was passaged into Erlenmeyer flasks (500 mL) containing a liquid medium (250 mL). The initial inoculum of *L. edodes*, weighing 0.1 g, was transferred to Oddoux medium and incubated at a temperature of 25 ± 2 °C in a natural photoperiod at 140 rpm on a shaker (ALTEL, Poland). To achieve a significant increase in the biomass of *L. edodes* for research, the mycelium from cultures grown in Erlenmeyer flasks (250 mL) was transferred to specially designed 10 L bioreactors, in which the volume of the medium was 8 L, and its mixing was ensured by the inflow of sterile air (40 L/min). During mycelium growth, carbon dioxide was removed through a sterile filter. A better growth of *L. edodes* biomass was observed in aerated liquid cultures on a modified Oddoux medium and on the same medium containing selenium in the form of selenite triglycerides at a temperature of 25 ± 2 °C in a natural photoperiod and during the 10-day growth cycle. Aeration and removal of carbon dioxide were found to be effective in optimizing the culture conditions, resulting in maximum mycelial growth within 10 days of cultivation compared to the control cultures without aeration (which took about 21 days to grow). The yield of biomass on the modified Oddoux medium was averaged to about 10.6 g dry weight (d.w.)/L, but with the addition of selenite triglycerides, the yield was about 15.1 g d.w./L. After 10 days of growth in the mycelium bioreactors, *L. edodes* were separated from the substrate, frozen, and dried by lyophilization (Freezone 4.5 freeze dryer, Labconco; temperature: −40 °C, UJ CM property).

To standardize the obtained selenium-enriched mycelium, the content of this element in the obtained material was determined by flame atomic absorption spectroscopy after prior sample mineralization (microwave mineralizer by ERTEC). Determinations were performed using a Thermo Scientific AAS3500 spectrometer (USA).

### 2.9. Statistical Analyses

The results were analyzed using STATISTICA 12 software (StatSoft Inc., Tulsa, OK, USA). A one-way ANOVA and a multiple range test were performed to determine the main effects of dietary treatments. A Tukey post hoc test was carried out to determine the differences between the groups, and the effects were considered to be significant at a probability level of *p* ≤ 0.05. For a comparison of the serum selenium concentration in the control and *L. edodes*/Se group, a *t*-test was used.

## 3. Results

The rearing parameters of calves are shown in Table 4. It was observed that nanosilica/E/OA/SB and *L. edodes*/Se additives did not affect the BW, average daily gain (ADG), FCR of the calves, or average daily feed intake (ADFI), except for average daily milk replacer intake (Table 4). It was lower at 35–42 d but increased significantly from 42 to 70 d in the animals receiving *L. edodes*. Blood parameters did not differ throughout the experimental period (Table 5). ALT and AST were not improved in the experimental groups compared to the control animals (Table 6). Similarly, the results obtained from hematoxylin–eosin staining of the intestinal and ruminal tissue did not differ between the experimental and control groups. The average intestinal length also did not differ (28 m in the control group, 27.5 m in the nanosilica/E/OA/SB group, and 26 m in the *L. edodes*/Se group; *p* > 0.82).

The serum selenium concentration at the beginning of the experiment did not differ between the control and experimental group (34.17 µg/L in the control group and 33.5 µg/L in the *L. edodes*/Se group). However, on days 35 and 70, statistically significant differences were observed in the concentration of the bioelement between the groups (44.33 and 51.33 µg/L in the control group, and 132 and 93 µg/L in the *L. edodes*/Se group, respectively; *p* < 0.001; Table 7).

There were no differences between control and experimental groups in morphometric indicators: villus height, villus width, and crypt depth in the duodenum and rumen of calves at 70 days of age. The results are shown in Table 8.

## 4. Discussion

In this study, the average BW of calves at birth did not differ between the groups (Table 4). Similarly, in later life, up to day 70, no statistical differences were noted. Throughout the rearing period, no effect was observed in the ADG, ADFI, feed intake, or FCR at 1–35, 35–42, and 42–70 days. We observed lower average daily milk replacer intake at 35–42 d, which later increased significantly from 42 to 70 d in the animals receiving *L. edodes*/Se. At 35 d of age, calves received starter; it is possible that they prefer to take forage over milk replacer and that palatability of the milk was slightly reduced due to the addition of *L. edodes*/Se. Later, however, this trend was completely reversed; the milk replacer intake was higher compared to the other groups. However, these results did not affect animal body weights or the milk replacer conversion ratio. No differences in milk intake were found between the control and nanosilica/E/OA/SB groups. This suggests that the additives did not affect the feed palatability. Microencapsulation of acidifiers allows for increased palatability of the forge by masking unpleasant taste of classical acidifiers [25]. A lack of significant differences in feed intake was also reported by Ribeiro [26] for nonencapsulated acidifiers. One study described the fact that supplementation of encapsulated acidifiers decreased ADFI of piglets; however, there were no differences in average daily gain ADG [27]. Spanghero [28] observed higher weight gain and a better feed conversion ratio in animals supplemented with encapsulated organic acids compared to the control. Dietary treatments did not affect the pH or the presence of pathogenic microbial strains, such as *Escherichia coli*, *Salmonella typhimurium*, and *Clostridium perfrigens*, in the feces. Feed acidification is supposed to intensively support GI functions in the postnatal period and improve BWG, ADG, and FCR in farm animals [3]. However, under the conditions of the experiment in the present study, it was not found to be beneficial. On the other hand, Chen [29] observed that the ADG of calves fed acidified milk was higher than in the control group between 7 and 180 days. The authors also noted a lower incidence of diarrhea and affected fecal bacterial diversity (especially of the *Bacteroides* strain). Several researchers have reported that dietary inclusion of organic acids improves growth rate and feed efficiency [2,30]. Encapsulated acidifiers were found to reduce the pH of the stomach and greatly decrease pH in jejunum and ileum of weaning piglets, and a significantly increased ratio between the villus height and crypt depth of jejunum was noted [31]. One of the most significant advantages of microencapsulation is that it can deliver organic acids along the whole intestine; therefore, we can expect to observe its effects in practically every part of the gastrointestinal tract [32]. Unfortunately, we did not observe any effect in the histometry of the rumen and duodenum.

Moreover, some studies [33,34] showed less occurrence of digestive disorders in animals, especially when they were fed ad libitum. A study by Bosi [35] reported that protected organic acids led to lower *E. coli* counts in the ileum and higher *Lactobacillus* counts in the colon, indicating that these acids are more effective in retarding the absorption of dietary acids and allowing more effective delivery of acids to the distal ileum, cecum, and colon. Variations in responses to dietary acidifiers observed in different studies may be related to differences in experimental methods, dietary ingredients that influence the natural production of acids, microbiota status, gastric pH, supplementation level of acidifiers, and age of animals [36].

In the present study, we aimed to evaluate whether pancreatic enzymes used with protected acidifiers could be more effective due to the lower pH of the GI tract. Guilloteau [6] noted that calves fed with milk replacer based on soy showed lower secretion of pancreatic enzymes, which caused a reduction in nutrient availability. Enzyme supplementation should improve digestive functions and allow for effective hydrolysis of feed components, resulting in higher BWG and better feed conversion. The hypothesis that enzymes may improve rearing parameters was proved incorrect by the findings of this study. It is likely that the availability of nutrients for the rumen and intestinal microbiota is changed, which may have an adverse effect on bacterial populations in the intestine, resulting in reduced production of VFAs. The second reason may be that pathogenic microorganisms have better access to amino acids that are necessary for their nutrition, which leads to their excessive proliferation. The last reason might be the fact that feed enzymes release antinutritional byproducts in the intestine, inhibiting the growth of commensal microbiota [37]. In this study, the third component of the additive used was butyrate, which belongs to the group of VFAs and is generally considered a stimulator of rumen development [38].

The present study did not show improved rearing parameters or better rumen development, which is in contrast to the results reported by Górka [39,40]. The addition of sodium butyrate to milk stimulated the development of the small intestine and eliminated the negative effects of milk replacer on rumen development. Guilloteau [6] also reported that the addition of sodium butyrate to calf diets had a positive effect on the development of the small intestine. The additive also supported intestinal cell proliferation, villus growth, and digestive enzyme activity, all of which positively affected calf performance and health [6]. The positive effect of a complex supplement at later time may be related to an improvement in the condition of the intestines, their faster maturation, and a decrease in inflammatory interleukins. Oral administration of sodium butyrate may significantly modulate the level of inflammatory cytokines in the ileum while having a less prominent effect on intestinal microbiota status [41].

Some nanoparticles (silver, titanium dioxide, fullerenes, zinc oxide, and magnesium oxide) have been proven to show antibacterial activity. The bactericidal action of nanoparticles is probably due to the inhibition of energy metabolism as a result of their internalization by the bacteria cell [42]. Certain doubts may arise from the fact that not all interactions with the intestinal microbiota have been studied, and the potential transfer of nanoparticles from the intestines of livestock to the foods of animal origin also remains unknown. To sum up, nanoparticles can be widely used in the feed of farm animals; however, further research is required to thoroughly understand their effects [43]. In the present study, we showed that the nanosilica particle-based additive did not influence the development of rumen and intestine in calves.

In this study, at the beginning of the experiment and later on, the serum selenium concentrations in the nonsupplemented group were below the reference values (33.5–34.17 µg/L). No improvement in rearing parameters was observed with the supplementation of *L. edodes*/Se, but the selenium level significantly improved. This suggests that the addition of modified *L. edodes* was effective in increasing this element in the animals’ blood. The findings of other studies confirmed that the organic form of selenium was better absorbed than sodium selenite [44,45].

In the present study, no significant treatment differences were observed in blood, biochemical, or histological parameters in the tested groups (Table 5). The groups were similar in terms of health status and showed no differences in morphological or biochemical indicators. A slightly elevated concentration of erythrocytes in the blood may indicate dehydration in animals. However, the obtained results showed that erythrocytes were slightly elevated (norms: 5–7 mln/mm^3^ [46]) in both control and experimental groups throughout the rearing period, which does not indicate other advanced disease processes related to the RBC system. Similar conclusions were drawn from the slightly lowered MCV. According to Winnicka [46], the level of thrombocytes should be 150–650 thousands/mm^3^, and an elevated level of thrombocytes may indicate iron deficiency, which should be monitored to ensure the good health status of animals and prevent thrombocytosis in all animal groups from 10 to 70 days of age. Elevated platelet counts may also be associated with inflammation in the body, especially in the case of farm animals [46]. Histometry analysis by Górka [40] indicated an increased length and width of rumen papillae in the group receiving sodium butyrate and no differences in the thickness of the muscle layer compared to the control. Similarly, Kotunia [47] highlighted the fact that milk replacers delay the maturation of the GI tract in piglets. The authors used sodium butyrate to improve mucosal development and observed a positive effect in the distal jejunum and ileum manifested by an increase in crypt depth, villi length, and mucosal thickness, whereas in the duodenum, the villi length and mucosal thickness were reduced. The conclusion that can be drawn based on the experimental results and the research cited above is that sodium butyrate used alone has a better effect on histometry of the gastrointestinal tract. It is possible that the remaining components affect the microbiota status or cell division within the intestinal villi responsible for their elongation. Nevertheless, this effect has no negative influence on the production parameters of calves.

## 5. Conclusions

The experiment conducted in the present work showed no beneficial effects of the two additives—nanosilica/E/OA/SB and *L. edodes*/Se—on rearing parameters, morphological and biochemical indicators, or rumen and ileum development in calves up to 70 days of life. However, dietary addition of *L. edodes*/Se significantly improved the serum selenium level in the *L. edodes*/Se group at 70 days compared to the control group. However, we observed lower and, later, significantly higher average daily milk replacer intake in animals receiving *L. edodes*, although it did not influence the other growing parameters or animals health.

## Figures and Tables

**Table 1:** Composition of one dose of the feed additive based on nanosilica.

Item	Content
Nanosilica, mg	3000
Protease, mg	18
Lipase, mg	45
Fat-coated organic acids (fumaric, malic, citric, and sorbic acid), mg	2000
Sodium butyrate, mg	10,000

**Table 2:** Nutritional value of milk replacer.

Item	Content of Ingredients
Crude protein (%)	20.5
Crude fat (%)	16
Crude fiber (%)	0.3
Vitamin A [IU]	25
Vitamin D_3_ [IU]	5
Vitamin E [mg]	300
Selenium [mg]	0.3
Copper [mg]	10
Iron [g]	90

**Table 3:** Composition and nutritional value of the starter (on DM basis) according to the DGL system [23].

Item	Composition of the Diet in %
Barley	20
Wheat	20
Corn	15
Oat	10
Soybean meal	21
Wheat bran	6.1
Chalk	1.6
Calcium phosphate	1.1
NaCl	0.4
Magnesium oxide	0.2
Vitamin–mineral premix	1
Molasses	3
Rapeseed oil	0.5
Item	Calculated Nutritional Value per kg of Feed
Energy [MJ EM, kg]	10.96
Crude protein [g]	180.01
Intestine digestible protein [g]	160.6
Ca [g]	9.06
P [g]	6.74
Mg [g]	2.74
Na [g]	1.89
Vitamin A [j.m.]	13
Vitamin D_3_ [j.m.]	3
Vitamin E [mg]	50
Vitamin K [mg]	1
Vitamin B_1_ [mg]	15
Vitamin B_2_ [mg]	4
Vitamin B_6_ [mg]	1
Vitamin B_12_ [mg]	0.02
Biotin [mg]	0.02
Nicotinic acid [mg]	20
Pantothenic acid [mg]	15
Folic acid [mg]	0.5
Mn [mg]	60
Zn [mg]	140
Fe [mg]	100
Cu [mg]	15
I [mg]	2
Se [mg]	0.5

**Table 4:** Effect of dietary supplementation with nanosilica and *L. edodes* mycelium on calves’ performance between 4 and 70 days (*n* = 6).

Item	Control ± SD	Nanosilica/E/OA/SB ± SD	*L. edodes*/Se ± SD	*p*
BW [kg]				
Birth	45.2 ± 4.44	45.7 ± 7.38	46.0 ± 6.99	0.96
7 days	45.1 ± 4.41	44.6 ± 5.13	44.8 ± 4.44	0.98
14 days	45.1 ± 4.40	45.3 ± 5.17	45.5 ± 4.82	0.99
21 days	46.8 ± 2.96	46.7 ± 5.50	46.7 ± 5.26	0.99
28 days	47.3 ± 4.04	48.8 ± 6.73	49.0 ± 6.87	0.86
35 days	50.4 ± 5.33	50.0 ± 7.22	50.5 ± 6.85	0.99
42 days	55.2 ± 5.44	55.2 ± 6.38	55.0 ± 6.25	0.99
49 days	59.4 ± 7.00	59.4 ± 6.10	58.9 ± 6.36	0.98
56 days	65.8 ± 9.25	64.9 ± 6.94	64.4 ± 7.48	0.95
63 days	71.7 ± 10.7	71.8 ± 7.56	71.4 ± 7.37	0.99
70 days	76.1 ± 12.77	77.5 ± 9.25	76.6 ± 9.86	0.97
ADG [g/d]				
1–35	148.51 ± 7.19	120.95 ± 15.54	127.78 ± 16.35	0.87
35–42	690.48 ± 25.15	740.48 ± 38.89	655.10 ± 34.60	0.6
42–70	744.64 ± 29.31	799.40 ± 19.28	770.92 ± 20.12	0.91
ADFI of the starter				
[g/d]	458.05 ± 29.89	471.05 ± 26.74	391.79 ± 21.34	0.86
35–42	1042.65 ± 54.88	1027.83 ± 35.31	898.18 ± 35.74	0.82
42–70				
Average daily milk replacer intake [g/d]				
5–35	483 ± 18.6	507 ± 16.1	499 ± 18.6	0.09
35–42	719 ^a^ ± 44.8	736 ^a^ ± 31.0	634 ^b^ ± 22.3	<0.001
42–70	538 ^a^ ± 63.2	520 ^a^ ± 57.0	690 ^b^ ± 23.5	<0.001
FCR [g/g]				
35–42	0.76 ± 0.59	0.73 ± 0.46	0.85 ± 0.90	0.95
42–70	1.52 ± 0.99	1.28 ± 0.32	1.14 ± 0.42	0.59
Milk replacer conversion ratio [g/g]				
5–35	3.99 ± 1.74	6.40 ± 1.91	6.3 ± 1.89	0.95
35–42	1.19 ± 0.51	1.32 ± 0.83	1.19 ± 0.66	0.94
42–70	0.85 ± 0.51	0.71 ± 0.16	0.91 ± 0.21	0.57

^a,b^—the values with different letters differ significantly (*p* < 0.001). Abbreviations: BW—body weight, ADG—average daily gain, ADFI—average daily feed intake, FCR—feed conversion ratio, nanosilica/E/OA/S—additive containing nanosilica with pancreatic enzymes; a mixture of fat-coated organic acids (fumaric, malic, citric, and sorbic acids) and sodium butyrate, *L. edodes*/Se—additive with *Lentinula edodes* enriched in selenium.

**Table 5:** Effect of dietary supplementation with nanosilica/E/OA/SB and *L. edodes* mycelium on hematological parameters at 10, 35, and 70 days of age (*n* = 6).

	Results at 10 Days of Age				Results at 35 Days of Age				Results at 70 Days of Age			
Item	Control ± SD	Nanosilica/E/OA/SB±SD	*L. edodes*/Se ± SD	*p*	Control ± SD	Nanosilica/E/OA/SB ± SD	*L. edodes*/Se±SD	*p*	Control ± SD	Nanosilica/E/OA/SB ± SD	*L. edodes*/Se ± SD	*p*
MCV (fl)	37.40 ± 2.70	38.33 ± 1.97	37.00 ± 2.00	0.58	33.17 ± 1.47	34.00 ± 1.41	33.33 ± 1.03	0.53	32.00 ± 1.26	32.83 ± 1.17	32.5 ± 1.22	0.51
MCH (pg)	12.10 ± 0.19	12.15 ± 0.58	11.7 ± 0.28	0.12	11.47 ± 0.3	11.48 ± 0.42	11.08 ± 0.58	0.24	11.40 ± 0.42	11.25 ± 0.57	11.25 ± 0.62	0.86
MCHC (g/dl)	32.40 ± 1.98	31.78 ± 1.26	31.5 ± 1.48	0.62	34.62 ± 1.97	34.03 ± 2.37	33.3 ± 2.49	0.62	35.58 ± 1.27	34.25 ± 1.55	32.5 ± 1.14	0.23
Erythrocytes (mln/mm3)	8.01 ± 1.17	7.91 ± 1.36	7.43 ± 1.16	0.76	7.62 ± 0.98	7.73 ± 0.84	7.11 ± 0.84	0.46	8.60 ± 0.94	9.18 ± 0.41	8.74 ± 0.58	0.33
Hematocrit (%)	0.30 ± 0.06	0.30 ± 0.06	0.27 ± 0.04	0.40	0.25 ± 0.04	0.26 ± 0.03	0.23 ± 0.03	0.60	0.28 ± 0.04	0.30 ± 0.02	0.28 ± 0.02	0.39
Hemoglobin (g/dl)	9.70 ± 1.54	9.63 ± 1.83	8.68 ± 1.34	0.48	8.73 ± 1.18	8.87 ± 1.04	7.88 ± 1.04	0.26	9.83 ± 1.23	10.33 ± 0.72	9.85 ± 0.83	0.60
Leukocytes (thousands/mm3)	8.84 ± 5.68	10.08 ± 3.46	11.05 ± 2.77	0.66	7.15 ± 0.93	7.93 ± 0.97	7.63 ± 3.15	0.79	8.45 ± 1.77	9.10 ± 1.12	8.05 ± 2.38	0.61
Thrombocytes (thousands/mm3)	846.00 ± 120.75	864.33 ± 87.37	900 ± 0.00	0.55	876.83 ± 42.67	900.00 ± 0.00	860.5 ± 63.79	0.33	845.83 ± 85.22	717.50 ± 146.14	716.83 ± 167.14	0.21
RDW (%)	17.62 ± 0.97	17.72 ± 0.69	17.8 ± 0.53	0.91	15.97 ± 1.65	16.02 ± 1.38	15.67 ± 0.98	0.89	14.35 ± 1.16	14.80 ± 0.75	14.8 ± 1.62	0.76
MPV (fl)	5.80 ± 0.27	5.83 ± 0.42	5.9 ± 0.42	0.89	7.20 ± 0.64	6.77 ± 0.98	6.9 ± 0.80	0.65	7.42 ± 0.43	7.03 ± 0.51	7.03 ± 0.59	0.42

Abbreviations: Nanosilica/E/OA/S—additive containing nanosilica with pancreatic enzymes; a mixture of fat-coated organic acids (fumaric, malic, citric, and sorbic acids) and sodium butyrate, *L. edodes*/Se—additive with *Lentinula edodes* enriched in selenium, MCV—mean corpuscular volume, MCH—mean corpuscular hemoglobin, MCHC—mean corpuscular hemoglobin concentration, RDW—red cell distribution width, MPV—mean corpuscular volume.

**Table 6:** Effect of nanosilica/E/OA/SB and *L. edodes* mycelium on the level of liver enzymes: ALT and AST (*n* = 6).

Item	Control ± SD (U/L)	Nanosilica/E/OA/SB ± SD (U/L)	*L. edodes*/Se ± SD	*p*
			(U/L)	
ALT				
10 days	4.83 ± 1.6	7.33 ± 5.68	8.00 ± 4.6	0.43
35days	4.83 ± 0.75	6.00 ± 3.85	6.17 ± 1.94	0.62
70 days	8.5 ± 4.14	9.17 ± 1.47	8.83 ± 2.14	0.92
AST				
10 days	23.66 ± 6.92	40.67 ± 27.81	27.00 ± 11.12	0.24
35 days	34.83 ± 4.49	37.67 ± 18.27	36.83 ± 5.91	0.91
70 days	43.00 ± 7.75	49.33 ± 5.75	46.67 ± 9.07	0.37

Abbreviations: ALT—alanine transaminase, AST—aspartate transaminase, nanosilica/E/OA/S—additive containing nanosilica with pancreatic enzymes; a mixture of fat-coated organic acids (fumaric, malic, citric, and sorbic acids) and sodium butyrate, *L. edodes*/Se—*Lentinula edodes* enriched in selenium.

**Table 7:** Effect of *L. edodes*/Se on serum selenium concentration (*n* = 6).

Item	Control ± SD	*L. edodes*/Se ± SD	*p*
Selenium [µg/L]			
10 days	34.17 ± 9.41	33.5 ± 8.34	0.89
35 days	44.33 ^a^ ± 3.93	132 ^b^ ± 28.56	<0.001
70 days	51.33 ^a^ ± 4.68	93 ^b^ ± 10.18	<0.001

^a,b^—the values with different letters differ significantly (*p* < 0.001). Abbreviations: Nanosilica/E/OA/S—additive containing nanosilica with pancreatic enzymes; a mixture of fat-coated organic acids (fumaric, malic, citric, and sorbic acids) and sodium butyrate, *L. edodes*/Se—additive with *Lentinula edodes* enriched in selenium.

**Table 8:** Effect of nanosilica/E/OA/SB and *L. edodes*/Se on the morphometric indicators of the duodenum and rumen of calves at 70 days of age (*n* = 3).

Item	Control ± SD	Nanosilica/E/OA/SB ± SD	*L. edodes*/Se ± SD	*p*
Duodenum				
Villus height (µm)	864.07 ± 126.75	865.43 ± 108.07	822.76 ± 149.66	0.12
Villus width (µm)	117.98 ± 31.14	119.18 ± 34.75	120.51 ± 30.25	0.99
Crypt depth (µm)	139.045 ± 45.55	144.89 ± 43.30	137.710 ± 43.23	0.63
Rumen papillae				
Height (mm)	2.47 ± 0.33	2.39 ± 0.34	2.54 ± 0.34	0.71
Width (mm)	0.34 ± 0.04	0.36 ± 0.04	0.33 ± 0.04	0.43

Abbreviations: Nanosilica/E/OA/S—additive containing nanosilica with pancreatic enzymes; a mixture of fat-coated organic acids (fumaric, malic, citric, and sorbic acids) and sodium butyrate, *L. edodes*/Se—additive with *Lentinula edodes* enriched in selenium.

## Data Availability

Data are available on request from the corresponding author.
